# Representativeness in health research studies: an audit of Greater Manchester Clinical Research Network studies between 2016 and 2021

**DOI:** 10.1186/s12916-023-03170-5

**Published:** 2023-11-29

**Authors:** Kathryn M. Abel, Maja R. Radojčić, Archie Rayner, Rabia Butt, Pauline Whelan, Isaac Parr, Lauren F. Gledhill, Ashley Minchin, Peter Bower, Holly Hope

**Affiliations:** 1 Centre for Women’s Mental Health, Division of Psychology and Mental Health, Faculty of Biology, Medicine and Health, University of Manchester, Manchester, UK; 2 Greater Manchester Mental Health NHS Foundation Trust, Manchester, UK; 3 National Institute for Health and Care Research Greater Manchester Clinical Research Network, Manchester, UK; 4 Centre for Health Informatics, Division of Imaging, Informatics and Data Science, Faculty of Biology, Medicine and Health, University of Manchester, Manchester, UK; 5 GM.Digital Research Unit, Greater Manchester Mental Health NHS Foundation Trust, Manchester, UK; 6 National Institute for Health and Care Research Applied Research Collaboration Greater Manchester, Manchester, UK

**Keywords:** Equity, Equality, Audit, Ethnicity, Representativeness, Health services

## Abstract

**Background:**

There are increasing concerns that participants in health research in the UK are not representative of the UK population, risking widening health inequities. However, detailed information on the magnitude of the problem is limited. Therefore, we evaluated if the health research conducted in the Greater Manchester region was broadly representative of its diverse population.

**Methods:**

We conducted an audit of all health  research studies conducted exclusively in Greater Manchester, using data from a national research network. Two researchers selected studies that were (1) an interventional or observational study of a health outcome; (2) ‘closed’ for recruitment between May 2016 and May 2021 and (3) human research. They extracted study information (dates, contacts, sample recruited, clinical speciality). Participant characteristics were sourced from published and unpublished manuscripts and requested directly from principal investigators and named study contacts.

Data were extracted, summarised and compared to the Greater Manchester population for the following metrics: ethnicity, sex, age, deprivation and smoking status. A weighted mean age estimate was calculated to account for variation in age reporting. Too few studies provided patient-level deprivation data so, using the area code of the recruitment site, the area level multiple deprivation, health deprivation and disability index and decile was derived. These data were geo-mapped using QGIS 3.26.

**Results:**

Overall, 145/153 (95%) studies met inclusion criteria and participant information was sourced for 85/145 (59%) studies, representing 21,797 participants. Participant information was incomplete for all metrics. Where ethnicity (*N* = 10,259) data were available and compared to Greater Manchester estimates there was evidence that ethnic minorities were under-represented (6% versus 16%). Most of the recruitment occurred in central Manchester (50%) and with NHS hospital settings (74%).

**Conclusions:**

Greater Manchester health research in 2016–2021 was centralised and under-represented ethnic minorities. We could not report which ethnic minority group was least represented because sourcing detailed participant information was challenging. Recommendations to improve the reporting of key participant characteristics with which to monitor representativeness in health research are discussed.

## Background

The primary aim of health research (e.g. trials of new products or interventions) is to improve health outcomes. The United Kingdom (UK) has a population of ~ 67 million, who are served by one of the largest universal healthcare systems in the world, the National Health Service (NHS) [1]. The UK’s current budget for NHS-led health research has reached over £250 million [2], indicating the importance of research for the health of a population and its economy. However, the value of health research is dependent on the generalisability of its findings to the wider population. It is increasingly acknowledged that the wider population may be underrepresented in and, thereby, underserved by research, leading to concerns about research equity [3].

Addressing inequity has important implications for how research is conducted. It requires structures that go beyond making health services simply available to all equally (health equality) and direct resources specifically so that they benefit everyone [4]. Most health research in England is supported by the National Institute of Health and Care Research (NIHR). In 2019, the UK Chief Medical Officers wrote: “…at the more applied, clinical and public health end of the (research) spectrum, there is a strong scientific need for research to be conducted with and in the populations most affected” [5] Therefore, the aim of this study is to evaluate if health research in Greater Manchester, a diverse region of England, is representative of the local population.

It is well-recognised that sociocultural, geographical and clinical factors all influence participation in health research; these factors also influence how people are likely to respond to new treatments or interventions and, thereby, the generalisability of research findings [6, 7]. For example, gender, age or ethnicity might influence how well an intervention works and change the effectiveness and cost-effectiveness of the intervention. Compared to the ‘real world’ patient population, clinical trial participants tend to be selected towards lower-risk, younger participants, and those without comorbidities; restricted sampling like this limits the external validity of trial results [8]. Significant geographical variation in recruitment for clinical studies in England, and recruitment that is not aligned with disease prevalence, are also important factors [3, 9]. However, a recent qualitative study reported that chief investigators of health research were reluctant to approach newer sites for recruitment, or sites with less previous or current research activity, deeming them ‘too risky’, and potentially compromising delivery of the trial to time and budget [10]. If this is the case, health research equity might be addressed by shifting research from the usual centres of excellence and conducting it in areas of highest health burden, population density and diversity [11]. Incentivising research in areas of highest health burden might address researchers’ concerns and be a pragmatic and efficient mechanism for increasing recruitment into studies.

In England, the NIHR Clinical Research Network (CRN) is a unique adjunct to the NHS, being an integrated research infrastructure covering all of England. It consists of 15 regional networks that support high-quality interventional and non-interventional health and care research [11]. The NIHR provided a recent estimate of the representativeness of funded randomised controlled trials (RCTs) between 2019 and 2021; whilst gender and ethnicity were broadly representative of the 2011 UK population, only 60% of studies reported ethnicity data and there was little consideration of socioeconomic or smoking data [12].

In this study, we aimed to extend this work by auditing participant recruitment into studies between 2016 and 2021 in Greater Manchester. Specifically, we examined all types of health research, and the characteristics of study participants compared to the local Greater Manchester population using the following demographic factors: age, sex, ethnicity, smoking status, deprivation level and geographical location. Greater Manchester is one of the most diverse areas in the UK with respect to wealth, sociodemographic status and ethnicity, making it an excellent setting to explore the representativeness of research.

We anticipated participant gender would be representative of that in the Greater Manchester population; and that the age of participants would represent the population requiring most healthcare (i.e. would be older than the mean Greater Manchester population). However, we also anticipated lower representation of ethnic minority groups and of socioeconomically deprived groups in health research. Our overarching aim is that these findings provide a baseline to inform and monitor how Greater Manchester can improve the representativeness and hence the value of its health research to the population.

## Methods

### Design and setting

This was an audit of all health research studies on the Clinical Research Network (CRN) portfolio in Greater Manchester, UK. NIHR portfolio studies must have ethical approval and be externally peer-reviewed and competitively funded, usually via research council, central government, commercial or non-commercial grants. The NIHR supports its portfolio via local CRNs that provide regional recruitment and delivery support. A portfolio study might receive support from more than one local CRN and recruit from multiple regions of the UK. However, our audit focussed on NIHR portfolio studies exclusively supported by the Greater Manchester CRN.

### Search strategy and selection criteria

The CRN runs an open, daily updated portfolio of supported research studies. After registration, the research community can search the Open Data Platform (https://public-odp.nihr.ac.uk/) and access study information. This dashboard allows filtering of studies based on study status, lead CRN and closure date.

Inclusion criteria for our study were: study recruitment in one or multiple places led by Greater Manchester-CRN; study ‘closed’ between May 2016 and May 2021; interventional or observational studies from any clinical speciality. Studies were excluded when more than one CRN supported the study, or there were no patient data, e.g. Delphi surveys, or developing techniques or methodologies. Two researchers identified studies on the portfolio and screened them for eligibility. For selected studies, they sought patient data initially by searching in Google Scholar for the study’s published/preprint research articles and for all types of published reports based on the study title, acronym and list of investigators. When a publication was unavailable, we contacted the study principal investigators directly by email and/or phone and requested baseline demographic characteristics of their study samples. In total, we made three attempts to contact investigators before the study was excluded. Finally, if, among assessed studies, the identified publication was a study protocol or did not include the required patient data, we emailed study investigators to provide these. We sent one reminder to these investigators before closing the study selection phase on 7^th^ October 2022.

### Data extraction and analysis

CRN Greater Manchester provided recruitment data for all included studies. These comprised the Greater Manchester NHS sites with the corresponding number of participants recruited. The number of recruited individuals does not always correspond to the study sample because recruited individuals could withdraw consent. Using these data, we created maps of health research activity across Greater Manchester in 2016–2021.

From the included studies, two researchers extracted information on sample size, age, sex, ethnicity, geographical location, deprivation and smoking status and created a dataset. The available data across studies were combined and pooled estimates were reported. We did not impute missing values. Age estimates were differently reported across the studies, either as a continuous variable with mean and standard deviation (SD), median and interquartile range (IQR), and range, or as a categorical variable with suitable categories. We described age as mean. When age median and range were reported, we used the Wan et al. estimation method [13] to calculate the mean from these and combine them with other studies that did report the mean and weighted for the study sample size. We did not report standard deviation as age estimates were sample-based, not individual-based. We reported frequencies of female or male sex and smoking status (‘currently’ smoking or not) variables. Few studies reported ethnic minority categories and those that did used inconsistent groupings that were challenging to combine into meaningful high-level categories. Due to this, ethnicity was categorised as ethnic minority or White background. We intended to retrieve any available individual-level indicators for deprivation; and to use postcode as an indirect index of deprivation.

Risk of bias usually judges the strength of the evidence based on the quality of the study and its design. This is not our focus here. We did not assess the quality of the included studies. Instead, we assessed if there was recruitment bias that might mean some groups are over/ underrepresented in health research.

To assess if our prevalence estimates were biased because a substantial number of studies by design selected on one of the characteristics of interest we excluded these studies, recalculated and presented the unbiased estimate for comparison. We conducted a sub-analysis and calculated raw and unbiased estimates by clinical speciality, presenting results for specialities with 500 participants or more. We also examined if studies that by design were more inclusive and did not select on one, two or three of the studied characteristics (age, sex and ethnicity) better approximated the Greater Manchester population.

Finally, we used Greater Manchester population estimates published by the Office for National Statistics (ONS) as a mid-2020 report [14]. The latest ethnicity data were available from the 2011 census [15]. We used the ONS Open Geography Portal (https://geoportal.statistics.gov.uk/) to obtain geographical data for Greater Manchester and the UK Government report of the Index of Multiple Deprivation (IMD) 2019 at the Lower layer Super Output Areas (LSOAs) [16]. LSOAs are small geographical areas with an average of 1500 residents (range 1000–3000) or 650 households [16]. The IMD 2019 is the official measure of deprivation based on seven weighted domains of deprivation calculated at an LSOA level, ranked nationally from most deprived (rank 1) to least deprived (rank 32,844), and expressed in deciles (decile 1 represents the most deprived 10% of LSOAs in England) [16]. Health Deprivation and Disability (HDD) is one of the IMD domains (weight 13.5%) that measures the risk of premature death and the impairment of quality of life through poor physical and mental health [16]. It is expressed similarly to IMD in deciles, where 1 presents the worst health deprivation. We used IMD and HDD deciles to visualise the deprivation in Greater Manchester relative to the position of Greater Manchester NHS Trust sites (based on coordinates) and CRN Greater Manchester recruitment for the included studies (based on LSOA) and within the boundaries of the ten administrative authorities (Bolton, Bury, Manchester, Oldham, Rochdale, Salford. Stockport, Tameside, Trafford, Wigan) that comprise Greater Manchester.

We used Python 3.10.7 and QGIS 3.26 to complete all data analysis and geographical visualisation.

## Results

### Study selection and characteristics

**Fig. 1:**
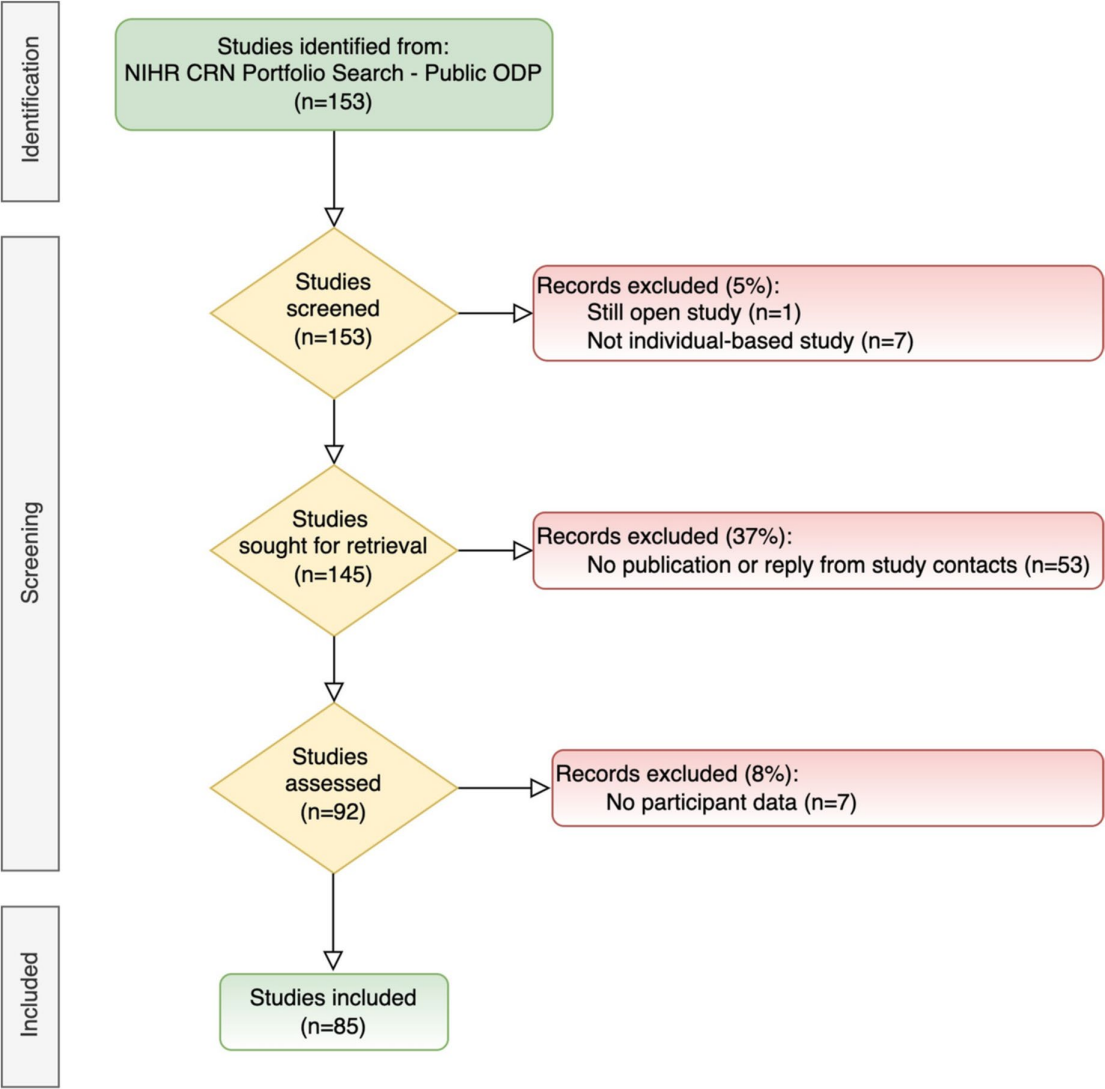
Selection of studies

We found 153 studies on the portfolio that were recorded as closed between May 2016 and May 2021 that were exclusively conducted in Greater Manchester. Of these, we excluded eight studies (5%); one study remained open and seven did not capture patient data. We sought participant demographic data from 145 studies. We could not obtain publications or responses from study contacts for 53 studies (37%). Of the remaining 92 studies, we excluded seven (8%), following two attempts to obtain these data from study contacts, because the publication was a protocol or did not include patient demographic data. Therefore, 85 studies were included in this report. Figure 1 shows the study flow diagram.

Included studies covered research within 18 clinical specialities; the most common were cancer (*N* = 16), mental health (*N* = 10), musculoskeletal disorders (N = 7), and dermatology (*N* = 6); equally common (*N* = 5) were dementia, metabolic and endocrine disorders, reproductive health and childbirth, and stroke; other specialities were represented by less than five studies.

### Data availability

Included studies had sample sizes ranging from 3 to 2952, comprising, a total of, 21,797 individuals (see Table 1). Ethnicity was available for 47.1% (*N* = 10,259), age 79.9% (*N* = 17,412), sex 88.6% (*N* = 19,322) and smoking status 46.9% (*N* = 10,226) of included individuals. Too few studies provided IMD quintile or alternative deprivation data, so we could not examine this further.

**Table 1:** Representatives of Greater Manchester population in Greater Manchester Clinical Research Network-led, single-site studies in 2016–2021

Characteristic	*Greater Manchester* *2021*	*Greater Manchester Clinical Research Network* *2016–2021*		Subset with unbiased estimate^a^	
Size, *N*	2,848,300	21,797		21,797	
Age (based on *N*, sample %)			17,412 (79.9)		16,909(77.6)
Mean	38.4	58.8		58.4	
Categories					
< 18 years	22.8				
18–65 years	61.3				
≥ 65 years	15.9				
Sex (N, sample %)			19,322 (88.6)		12,651(58.0)
Women, %	50.7	66.3		49.3	
Ethnicity (N, sample %)			10,259 (47.1)		10,106(46.4)
White, %	83.8	93.3		93.7	
BAME, %	16.2	6.7		6.3	
Asian, %	10.1				
Black, %	2.8				
Mixed, %	2.3				
Other, %	1.0				
Smoking (*N*, sample %)			10,226 (46.9)	NA	
Currently smoking, %	15.0	33.6			
Quintile of deprivation (*N*, sample %)			19,352 (90.1)	NA	
Most deprived 1	40.3	17.7			
2	21.5	45.8			
3	13.4	33.3			
4	13.6	0.0			
Least Deprived 5	11.3	3.1			

Greater Manchester population estimates are from the Office for National Statistics mid-2020 report, except ethnicity which is based on the census 2011 report. Unbiased estimates exclude studies that selected on that characteristic. ^a^No studies sampled on smoking status

### Participant representativeness

Compared to the Greater Manchester population, fewer people who took part in Greater Manchester health research were from ethnic minorities (5.7% versus 16.2%). Three studies were selected on ethnicity (one selected white participants only and two selected Afro-Caribbean participants, *n* = 153), but the exclusion of these studies did not alter participation prevalence (see Table 1).

The estimated weighted mean age of participants was 58.8 years compared to the Greater Manchester mean age of 38.4 years. Nine studies selected on age (2 only children and 7 only older adults *N* = 723), but the unbiased age estimate was unaffected by their exclusion.

Two-thirds (66.3%) of participants were women; however, 21 studies (*N* = 6671) had a focus on a single sex — 19 selected only women and 2 only men. When single sex studies were removed 50% of participants were women, which was comparable to the Greater Manchester population.

Twice the number of participants (33.6%) reported current smoking than the Greater Manchester population (15.0%). No studies selected on this characteristic.

The following clinical specialities had a total sample size greater than 500 and were examined for participant representativeness: cancer, mental health, musculoskeletal disorders, primary care, renal disorders and respiratory disorders (Table 2). Participants from ethnic minority groups were underrepresented in all clinical specialities. Age varied in accordance with clinical speciality. For example, mean age of participants in mental health research was 31.2 years whereas in physical health research, mean ages were over 50 years. The high prevalence of women in Greater Manchester research was driven by female cancer studies (82.5% women). Smoking rates were notably high among studies of renal disorder (64.6%).

**Table 2:** Sample characteristics of Greater Manchester Clinical Research Network-led, single-site studies by clinical speciality

Clinical Speciality	Age				Women				Ethnic minority group				Smoking^a^	
	All		Unbiased		All		Unbiased		All		Unbiased		All	
	Total *N*	Mean	Total *N*	Mean	Total *N*	%	Total *N*	%	Total *N*	%	Total *N*	%	Total *N*	%
Cancer	8637	58.6	8014	58.6	8637	82.5	3106	52.0	3835	6.3	3835	6.3	5583	20.5
Mental Health	626	31.2	432	31.2	626	38.2	500	38.6	432	NP	432	NP	194	NP
Musculoskeletal Disorders	605	56.3	605	56.3	605	66.6	605	66.6	NR	NR	NR	NR	482	NP
Primary Care	3530	59.1	3433	59.1	3053	57.8	3467	57.0	1306	2.4	1306	2.4	NR	NR
Renal Disorders	3697	66.1	3073	66.1	1223	39.8	2972	37.8	3053	4.7	3053	4.7	3053	64.6
Respiratory Disorders	667	63	22	63	667	96.3	49	NP	645	2.8	645	2.8	667	12.4

Unbiased estimates exclude studies that selected on that demographic. *NA* not applicable, *NR* not reported, *NP* not presented. Total *N* < 500.

^a^No studies sampled on smoking so unbiased estimated not calculated

### Assessment of selection bias

There was considerable attrition in the number of studies with data that did not select by design; 60 studies (*N* = 16,909) did not select on age, 43 studies did not select on age or sex (*N* = 11,476) and 20 studies (*N* = 5218) did not select on age, sex and ethnicity. As studies became less selective the mean age of the participant and smoking prevalence increased, the prevalence of female participants decreased, whilst the prevalence of ethnic minority participants was stable (Table 3).

**Table 3:** The variation in sample characteristics as the sampling strategy becomes less biased

Unbiased estimates	Age		Women		Ethnic minority group		Smoking^a^	
	Eligible *N*	Mean	Eligible *N*	%	Eligible *N*	%	Eligible *N*	%
Age	16,909	58.4	16,909	65.4	9037	6.2	9307	34.2
Age + sex	11,476	60.2	11,475	49.8	5307	6.8	6026	48.0
Age + sex + ethnicity	5218	64.8	5218	43.5	5218	6.3	2956	65.7

Unbiased estimates exclude studies that selected on that characteristic.

^a^No studies sampled on smoking

### Recruitment site representativeness

Greater Manchester area code information was available for 83 (97.6%) studies including 19,532 (90.1%) individuals. Two-thirds of the studies recruited from one study site in Greater Manchester, whilst the maximum number of sites (healthcare institutions in Greater Manchester) was 16. 17.7% of participants in Greater Manchester studies were recruited at sites situated in areas of high deprivation (1st quintile of deprivation), whereas 40.3% of the Greater Manchester population live in areas of high deprivation (Table 1). Administrative areas where more than 40% of the population live in areas of high deprivation include Bolton, Manchester, Oldham, Rochdale, Salford and Tameside whilst Trafford and Stockport are the least deprived boroughs.

**Fig. 2:**
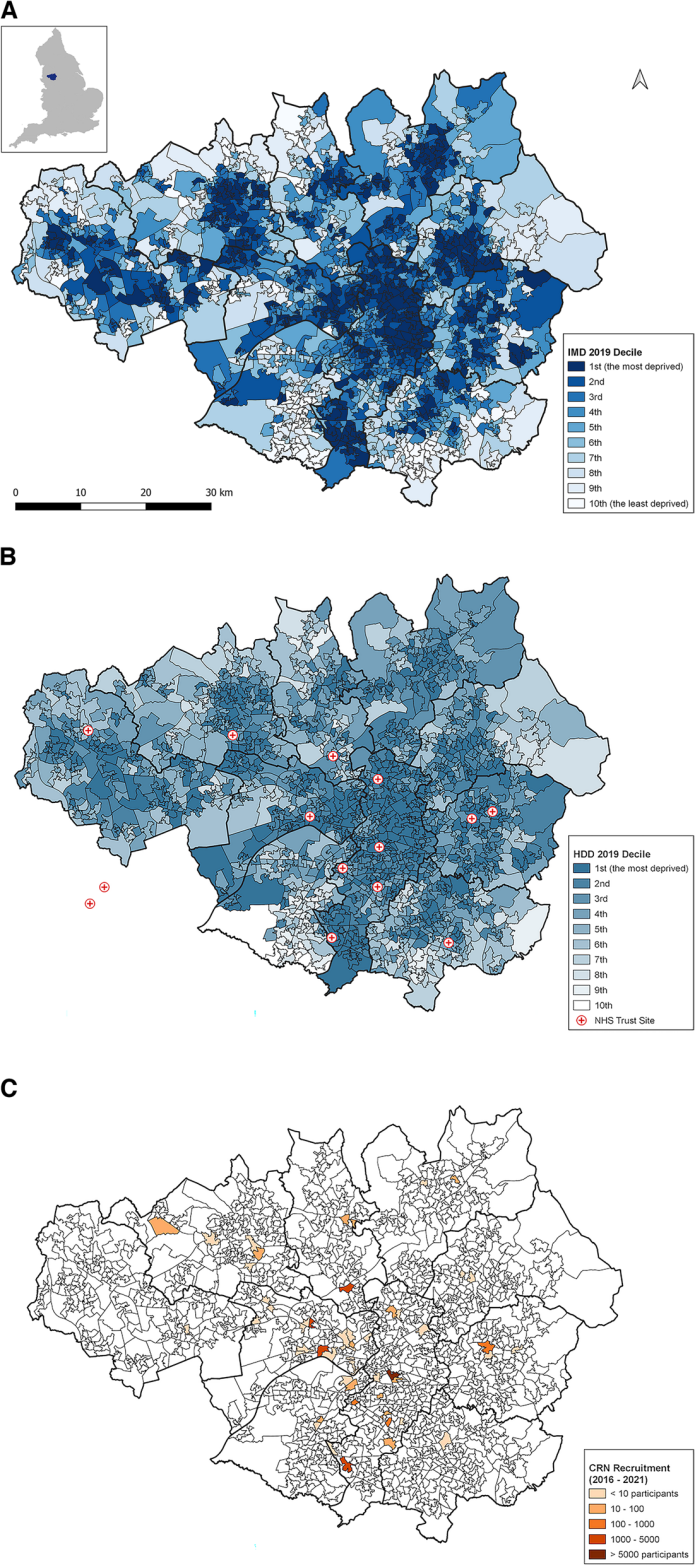
Map of deprivation and participant recruitment by Lower layer Super Output Area (LSOA) across Greater Manchester (Greater Manchester). **a** Greater Manchester location in England (upper left corner) and distribution of the Index of Multiple Deprivation (IMD) 2019 Decile in Greater Manchester. **b** Distribution of the Health Deprivation and Disability (HDD) 2019 (one of seven IMD domains) with location of 14 Greater Manchester National Health System (NHS) Trust sites (two sites are geographically in Cheshire County). **c** Distribution of participant recruitment sites for the included studies between 2016 and 2021

Figure 2a shows the distribution of the IMD decile, and Fig. 2b the distribution of the HDD decile for the NHS trusts in Greater Manchester. Most of the recruitment sites were NHS hospital trusts, situated in deprived areas.

Figure 2c shows recruitment across Greater Manchester. Overall, three-quarters (74.2%, *N* = 14,500) of individuals were recruited at NHS trust sites. Most of the recruitment was completed in Central Manchester (*N* = 9809), Salford (*N* = 7992) and Bury (N = 1118), accounting for 96.8% of participants. The Central Manchester University Hospitals NHS Foundation Trust was involved in recruitment for 42 studies, and the Salford Royal NHS Foundation Trust for 25 studies. Over 5 years, 2016–2021, healthcare institutions in Tameside recruited 517 and in Bolton, Oldham, Rochdale, Stockport, Trafford and Wigan 104 participants collectively from a population of 1.5 million.

## Discussion

We audited all single-site, NIHR-supported and Greater Manchester-CRN-managed studies that had closed to recruitment between 2016 and 2021, describing the key characteristics (ethnicity, age, sex, and social class/smoking status) of recruited subjects. We report that 6% of samples included participants from ethnic minority backgrounds compared to the proportion of ethnic minorities in the Greater Manchester population (16%). This underrepresentation was replicated across clinical speciality and when studies which specifically selected on participant characteristics were excluded. Overall, study samples provided a good representation of the Greater Manchester population in terms of sex. After removing single sex studies, 50% of study participants were women. As we anticipated, the participant population was older than the mean Greater Manchester population. We also report that most of the participant recruitment took place in centrally located Greater Manchester NHS Trust sites where centres of clinical excellence and research tend to be located.

### Research in context

Other contemporary reviews of representation in research also reported that women were adequately represented, but other groups were underrepresented. In a review of 213 Pfizer randomised controlled trials in the USA, participation was at or higher than US census levels for women and for Black or African American groups, but Hispanic or Latino, Asian, Native Hawaiian and Pacific Islander, American Indian, Alaska Native participants were underrepresented [17]. A recent evaluation of a large US biomedical research programme “All of Us” reported that, compared to US and state referents, non-Hispanic Black or African American groups were overrepresented whilst Hispanic and Latino, non-Hispanic Asian and multiracial groups were underrepresented. “All of Us” aims to recruit inclusively and equitably, to that end the authors insisted more should be done to over-recruit in order to redress the historic underrepresentation of certain groups in US health research[18]. In a study of the representativeness of 11 perinatal mental health studies conducted during the COVID pandemic, Black, Indigenous and other races and ethnicities were underrepresented despite the known racial and ethnic health disparities in pregnancy. Compared to our audit, there was detailed reporting of race and ethnicity in these studies. UK research may have a particular problem. Authors of a recent systematic review of the representativeness of 30 UK COVID vaccine trials reported limited and opaque reporting of participant ethnicity, similar to that found here, meaning it was difficult to be certain to what extent Asian, Black, Mixed and other ethnic minority groups were underrepresented in these trials [19].

### Strengths and limitations

There are several approaches to exploring the representativeness of patient recruitment. Some studies focus on a specific disease of interest and compare study participants with the characteristics of the general population with the same disease of interest. For example, a recent systematic review of 224 perioperative medicine trials reported that age exclusions and sampling bias meant the studied population were younger than the clinical population [20]. This approach addresses representativeness within a clinical speciality where age differences are easier to interpret. The approach we have taken here is complementary. Comparing the characteristics of patients recruited to research within the wider Greater Manchester population is complex when characteristics are clearly health related — such as age. However, there are characteristics where comparison is easier to interpret. For example, although there are some examples where recruitment of specific ethnic groups is indicated (e.g. sickle cell disease), this is not generally true. Our finding, that rates of ethnic minority participation are lower than the general population average, is of note. Although there are challenges in interpretation, our approach allows an evaluation of the totality of health research being conducted within a specific region, rather than a specific (and more limited) subset of research activity. Both are likely to be important to patients, those who commission health care, policy makers and funders.

Conducting this audit in Greater Manchester is a strength because, outside of Birmingham, this is the most diverse region in England. Access to the well-maintained CRN research dashboard improved the quality and content of the audit, particularly the ability to map recruitment. However, significant limitations remain. We were unable to obtain participant information for 53 out of 145 studies, accounting for ~ 4000 participants; therefore, it is possible there were studies with more or less diverse populations and their exclusion might change our overall findings. Ethnicity and social class measures (deprivation and smoking status) were less well reported and we had insufficient data to report high-level ethnic minority categories with confidence or index of deprivation at the patient level. Using recruitment site rather than participant address, means the data are not truly representative of where patients live. Social class and deprivation are important indicators of inequity because morbidity and severity of disease are overrepresented, and health research participation is underrepresented, in the most deprived areas and lowest social class. We were only able to use the IMD at the level of the recruitment centre. Although this gave us some measure of the aggregate deprivation of an area where the recruitment site was located, it did not tell us that people living in poverty in these areas of deprivation were taking part in that research. Therefore, to try to overcome this problem, we used smoking status as a patient-level indicator of social class. Not only is smoking often reported in health research, but in recent years, since smoking in the population has become less common, it acts as a relatively good indicator of social class [21]: rates of smoking increase with level of deprivation (31% in the poorest decile versus 9% in wealthiest) [22]. We report that most of the recruitment occurred within NHS hospital institutions located in central city locations which tend to sit in more deprived areas, and 33.6% reported smoking. However, the most common type of clinical research was cancer, meaning we should expect elevated levels of smoking in the sample population; we might interpret the high proportion of *non-smoking* in this cohort as evidence that most of the sample population are from wealthier areas than where they were recruited. Ideally, we would have used patient-level area code data to calculate IMD and describe the wealth distribution of the sample.

### Future implications and recommendations

These data, and the recent NIHR audit aggregating data across 140 RCTs conducted between 2019 and 2021 [12], provide valuable insight into the representativeness of funded clinical studies in the UK. Research participation provides advantages both to patients and healthcare services [23]. However, if clinicians do not consider the evidence to be generalisable to their clinical patient population, they might not offer an intervention [6, 10].

This study highlights significant gaps in the reporting of basic information about participants in clinical studies — especially social class, smoking status and ethnicity. These elements will be important if we are to address the challenges of inclusion and equity in research that are prioritised in the new CRN contract from 2024 [24].

The CRN supports participants to access and take part in research; and healthcare institutions and researchers to recruit participants for studies. A key feature of ‘Open for Business’ (UK Government strategies post Brexit) is to encourage inward investment from industry to undertake their research (Pharma and Medtech) in the UK market [25, 26]. High-quality science, rich NHS data and patient resources are key aspects of the offer. Delivering inclusion in studies is vital if findings are to generalise to the wider population and deliver improved outcomes. Individual-level data on sample diversity and levels of deprivation, therefore, must be improved in these datasets; geo-mapping could be used to identify and monitor the representativeness and equity of samples. Different healthcare levels, and non-centrally located institutions, should be adequately supported to exploit their potential to address inequity by undertaking research in areas of high disease burden [10].

It was challenging to reach investigators and obtain even the most basic summary information needed to ascertain representativeness. Equitable research should start with monitoring and transparent reporting of participant characteristics during recruitment. Obtaining data from studies once closed is challenging. We recommend that CRN support should be predicated on a minimum reporting of participant characteristics from the study outset. To make this as easy and secure as possible, CRNs could request non-identifiable participant data in a specific format, to be converted automatically to metrics that allow fair comparison and monitoring over time.

Digital tools can support equity monitoring of clinical studies. We are currently scoping the development of an in-study digital tool to monitor participant equity within the CRN’s open data platform through the MRC DATAMIND mental health informatics programme [27]. The DATAMIND equity audit tool will enable routine monitoring and reporting of recruitment patterns of clinical studies. The digital tool can support research teams to plan for more equitable recruitment by clearly identifying specific underserved groups. Other digital approaches have used machine learning to quantify and visualise gaps in the representativeness of clinical studies [28].

We think our approach is potentially translatable to other regions or countries where health research is undertaken, although it is dependent on good data collection on research activity at a regional level, as our ability to conduct this audit was dependent on online access to the majority of health research conducted in England. This is a unique advantage, and we are not sure if there are other equivalent examples. All health systems will have to deal with these issues as they arise within their own regions and administrative authorities. We believe representation cannot be tackled if it is not monitored over time. We hope the recommendations made serve as a model for how representation in research can be addressed.

## Conclusion

In conclusion, we report that health research in 2016–2021 in Greater Manchester, one of the most diverse English administrative authorities, was centralised and not representative of the diverse local population. This audit serves as a useful benchmark against which to assess both the representativeness of study participation and the collection and reporting of clinically relevant patient characteristics. It also serves as a baseline against which to monitor future improvements.

## Data Availability

Original study information can be accessed by eligible researchers and clinicians via the NIHR CRN Portfolio Search — after registration and acceptance onto Open Data Platform (ODP) (https://public-odp.nihr.ac.uk/). The data used in this specific study are available from: github.com/HollyHope/CRN_Audit.
